# Looking at the bigger picture: effect of performance-based contracting of district health services on equity of access to maternal health services in Zambia

**DOI:** 10.1093/heapol/czz130

**Published:** 2019-10-29

**Authors:** Collins Chansa, Mulenga Mary Mukanu, Chitalu Miriam Chama-Chiliba, Mpuma Kamanga, Nicholas Chikwenya, Ben Bellows, Naasegnibe Kuunibe

**Affiliations:** Heidelberg Institute of Global Health, Heidelberg University, Im Neuenheimer Feld 130.3, 69120 Heidelberg, Germany; Department of International Research and Development, American Institutes for Research, 2nd Floor, Elunda 2, Rhodes Park, Lusaka, Zambia; Institute of Economic and Social Research, University of Zambia, Plot No. 2631 Chudleigh, PO Box 30900, Lusaka, Zambia; Department of Policy and Planning, Ministry of Health, Ndeke House, Haile Selassie Avenue, PO Box 30205, Lusaka, Zambia; Department of Transport, Ministry of Transport and Communications, Fairley Road, PO Box 50065, Lusaka, Zambia; Population Council, 4301 Connecticut Avenue, Suite 280, Washington, DC 20008, USA; Heidelberg Institute of Global Health, Heidelberg University, Im Neuenheimer Feld 130.3, 69120 Heidelberg, Germany; Department of Economics and Entrepreneurship Development, University for Development Studies, Wa Campus, Upper West Region, Ghana

**Keywords:** Performance-based contracting, contracting, purchasing, results-based financing, maternal health, segmented regression analysis, Zambia

## Abstract

Zambia has been using output-based approaches for over two decades to finance whole or part of the public health system. Between 1996 and 2006, performance-based contracting (PBC) was implemented countrywide with the Central Board of Health (CBoH) as the provider of health services. This study reviews the association between PBC and equity of access to maternal health services in Zambia between 1996 and 2006. A comprehensive document review was undertaken to evaluate the implementation process, followed by a trend analysis of health expenditure at district level, and a segmented regression analysis of data on antenatal care (ANC) and deliveries at health facilities that was obtained from five demographic and health survey datasets (1992, 1996, 2002, 2007 and 2014). The results show that PBC was anchored by high-level political support, an overarching policy and legal framework, and collective planning and implementation with all key stakeholders. Decentralization of health service provision was also an enabling factor. ANC coverage increased in both the lower and upper wealth quintiles during the PBC era, followed by a declining trend after the PBC era in both quintiles. Further, the percentage of women delivering at health facilities increased during the PBC era, particularly in rural areas and among the poor. The positive trend continued after the PBC era with similar patterns in both lower and upper wealth quintiles. Despite these gains, per capita health expenditure at district level declined during the PBC era, with the situation worsening after the PBC era. The study concludes that a nationwide PBC approach can contribute to improved equity of access to maternal health services and that PBC is a cost-efficient and sustainable policy reform. The study calls for policymakers to comprehensively evaluate the impact of health system reforms before terminating them.

**Key Messages**

- Performance-based contracting can contribute to a health system-wide improvement in equity of access to maternal health services.
- High-level political support, a legal framework and strategic planning are essential for a robust and functional process of contracting and managing health services.
- Policymakers should comprehensively evaluate the effect of health system reforms before reorganizing the financing and implementation arrangements.

## Introduction

There is wide recognition that provision of health services through the public sector is characterized by significant inefficiencies and failure (Liu *et al.*, 2004). This can be attributed to lack of incentives to perform optimally and to deliver appropriate health services for the people. Consequently, a number of countries have over the past four decades sought to improve purchasing and management arrangements in the public sector by contracting-out primary healthcare services (Liu *et al.*, 2004; Evans, 2006). The justification is that the state is overstretched and that it should stimulate competition by allowing the private sector or autonomous public sector entities to provide public health services (Mills, 1998; Palmer, 2000; Liu *et al.*, 2004). This line of argument complements the ‘New Public Management’ thinking which advocates that governments should move from a concern to do, towards a concern to ensure that things are done or from rowing to steering (Travis *et al.*, 2002). It is envisaged that if governments assume the stewardship role and introduce market-led mechanisms [including performance-based contracting (PBC)] into the public health system, there would be greater transparency, accountability and consumer responsiveness, efficiency, quality and equity (Abramson, 2004; Liu *et al.*, 2004).

By definition, contracting is ‘a purchasing mechanism used to acquire a specified service, of a defined quantity and quality, at an agreed-on price, from a specific provider, for a specified period’ (Taylor, 2003, p. 158). Abramson (2004) further provides two broad options for contracting within which all types of contractual arrangements fall, i.e. contracting-in and contracting-out. Contracting-in takes place when a higher level of government (i.e. the central Ministry of Health) contracts with a lower level of government (i.e. a region, province, district or health facility) to manage and/or deliver specific health services within a public sector context (Abramson, 2004). On the other hand, contracting-out happens when a private entity is engaged to deliver specified health services in the public sector in exchange for payments (Abramson, 2004).

Since the advent of PBC in the 1980s, a number of developed and developing countries have implemented some form of contractual arrangements with public and/or private entities for management or delivery of specific clinical or non-clinical services (Mills and Broomberg, 1998; Abramson, 2004; Liu *et al.*, 2004). In Africa, Zambia was the first country that contracted-in the management of the entire primary healthcare services to an autonomous public organization (Lake and Musumali, 1999; Bossert *et al.*, 2003). This was achieved in 1996 with the establishment of the Central Board of Health (CBoH) as an implementing agency through a provider-purchaser arrangement (Bossert *et al.*, 2003). Establishment of the CBoH was part of the health reforms which commenced in 1991 aimed at decentralizing health service provision by ensuring that all Zambians have ‘equity of access to cost-effective quality health care as close to the family as possible’(Ministry of Health, 1991). The main argument for establishing the CBoH was premised on both economic and political principles aimed at achieving better health outcomes by instilling a performance culture, motivating the public sector health workforce to perform better, expanding coverage of health services, improving quality of care (Ministry of Health, 1991; Kalumba *et al.*, 1994); and reducing political interference with public health service delivery by establishing autonomous health boards (Kalumba *et al.*, 1994).

**Figure 1:**
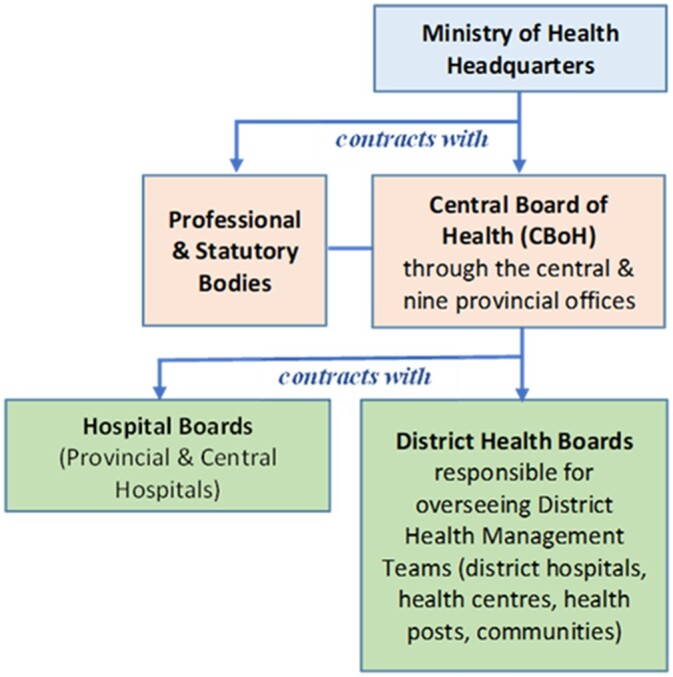
Contractual arrangements: Zambia Public Health System (1996−2006). *Source with modification:*Kalumba (1997).

As part of the implementation arrangement, the CBoH assumed the role of provider of health services while the Ministry of Health became the purchaser with its functions limited to policy formulation, setting of operational and clinical guidelines, and regulation (Ministry of Health *et al.*, 1997). In addition, autonomous health management boards were created in all the existing 72 districts through which the CBoH signed performance contracts for the provision of primary healthcare services as outlined in the Basic Health Care Package (Figure 1). A process of delinking health workers was also initiated to allow for all health workers to be directly employed by District Health Boards instead of the public civil service (Kalumba *et al.*, 1994; Lake and Musumali, 1999).

Despite the use of PBC, some policymakers argued that coverage and outcome indicators did not improve during the period 1996–2006 (Ministry of Health, 2004a). As a matter of fact, several studies that have evaluated the impact of PBC in low- and middle-income countries have found mixed results (Mills and Broomberg, 1998; Preker and Harding, 2003; Liu *et al.*, 2007; Lagarde and Palmer, 2009). In particular, there is limited evidence on the health system-wide effects of PBC whereas its impact on equity, quality and efficiency is largely unknown (Liu *et al.*, 2007). This is because most of the studies on PBC have focused on specific health diseases and conditions (i.e. childhood illnesses and malnutrition), primary healthcare services (reproductive, maternal and child health; Liu *et al.*, 2004), diagnostic and laboratory services, acute and long-term hospital care, and non-clinical services such as catering, cleaning, laundry, maintenance of medical equipment, storage and distribution of medicines, etc. (Mills and Broomberg, 1998). There are very few studies that have looked at the effect of PBC at the entire health system or national level.

In the case of Zambia, lack of evidence on the impact of PBC and other contextual factors contributed to the dissolution of the CBoH in 2006. However, in 2007—a year after the dissolution of the CBoH—Zambia recorded notable improvements in maternal and child health coverage and outcome indicators as observed in the 2007 Zambia Demographic and Health Survey. This raises questions as to whether the gains which were observed were due to the immediate impact of dissolving the CBoH or lagged benefits from a decade of implementing PBC. This article reviews the association between PBC and equity of access to maternal health services in Zambia over the period 1996−2006 when the CBoH was in place.

## Methods

The study reviews three aspects: implementation process, level of spending and coverage of maternal health services. Although the PBC era was 1996−2006, the period 1987−2014 was analysed in order to establish the trend before, during and after PBC. To facilitate the analysis, a mixed-methods approach—that combined qualitative and quantitative research techniques was used. This approach is commonly used and recommended by a number of scholars as it enables a researcher to gain access to a variety of insights, to challenge and verify perceptions, and to form a broad basis on which to make conclusions (Creswell *et al.*, 2011). Thus, the qualitative component of the study involved a comprehensive document review to gather information on the implementation process. For the quantitative part, data on district health expenditure and maternal health were collected and analysed.

### Document review

A comprehensive review of policy documents, strategic plans, independent studies and research articles which were produced in the early 1990s, 1994−1999, 2000−2005 and 2007−2014 was undertaken. The documents were carefully selected to acquire information on the historical thinking and motivation to introduce PBC (Ministry of Health, 1991); reform agenda, key institutions and implementation arrangements, and processes (Kalumba *et al.*, 1994; Kalumba, 1997); and empirical evidence on the successes and challenges over time (Ministry of Health *et al.*, 1997; Lake and Musumali, 1999; Ministry of Health, 2000, 2004a,b; Bossert *et al.*, 2003; Chansa, 2009). Furthermore, district action plans, budgets and expenditure reports; planning handbooks; service contracts; and minutes and action taken reports from the health sector committee meetings were also reviewed. These rich data allowed us to track and assess the quality of the contracting and implementation process during the period under review.

### District health expenditure dataset

We analysed district-level health expenditure data through the public health system over the period 1995–2014 aimed at establishing the level of expenditure before, during and after the intervention period. These data were obtained from the Ministry of Health and comprises operational (recurrent) expenditure^1^ from both the Zambian government and external development partners to the district ‘basket’ which was subjected to annual action planning and budgeting. To adjust for inflation, the gross domestic product deflator (base year = 2010) was used. This is because Zambia moved to the base year of 2010 in 2014 in line with the 2010 economic census (Central Statistical Office, 2014). Total district recurrent per capita health expenditure was calculated by dividing the total annual district-level recurrent expenditure by the total annual national population. The annual population figures were obtained from the Central Statistics Office covering the period 1995−2014.^2^

### Demographic and health survey datasets

Raw data on maternal health was accessed from five demographic and health surveys (DHS) for the years 1992, 1996, 2002, 2007 and 2014. DHS is a cross-sectional household survey that is conducted every 4–5 years and provides nationally representative coverage and outcome data on demography and population health status. Detailed information on the DHS methodology is available on the DHS website at https://dhsprogram.com/data/available-datasets.cfm. Individual recode STATA datasets from the DHS were used to aggregate data on two maternal health variables of interest, namely: antenatal care (ANC) and deliveries at health facilities (Table 1). These two indicators were among the key priority health indicators which were being monitored by the government during the period under review aimed at improving maternal and child health. To incorporate quality, only data on pregnant women who had at least four ANC visits at the stipulated times during their pregnancies were included. The resulting pooled dataset covers the period 1996–2014 for ANC and 1987–2014 for delivery care.

**Table 1:** Treatment variables, definitions, number of observations and time period

Treatment variable	Calculation	Observations analysed	Time period
Proportion of women who had four or more ANC visits at stipulated times during their last pregnancy preceding the survey^a^	Numerator: Total number of women with at least four visits at stipulated times	17 861	1996–2014
	Denominator: Total number of women with a birth per year		
Proportion of deliveries at health facilities	Numerator: Total number of births delivered at a health facility in a year	40 127	1987–2014
	Denominator: Total number of births per year		

a During each ANC service, woman are supposed to receive the following services: blood pressure checked, blood sample taken, urine sample taken, iron tablets/syrup given, fansidar (anti-malarial drug) taken and tetanus injection.

#### Preparation of DHS data before preliminary analysis

The main steps in preparing the dataset for preliminary analysis included identifying the variables of interest in the datasets from the five DHS, reshaping the dataset from wide format to panel format, categorizing covariates and outcomes of interest into yearly intervals and merging the datasets from the five DHS into one pooled dataset. Each outcome variable was expressed as a proportion (Table 1) and stratified by wealth quintiles (bottom two and upper two quintiles). For 1992, 1996 and 2002, an additional step involved merging the individual recode dataset with the wealth index dataset because the individual recodes datasets did not have the wealth variable. For the 2007 and 2013/14 DHS, wealth variables were available. A summary of the outcome variables, definitions, number of observations and time periods are shown in Table 1.

**Figure 2:**
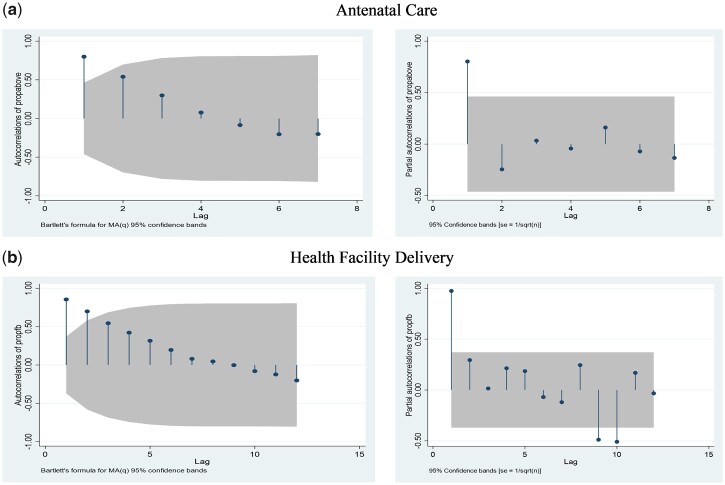
Test for autocorrelation and partial autocorrelation. *Source:* Authors’ construction from DHS data.

Data were inspected for presence of wild points, linear trend and seasonal patterns by plotting the outcome variable against time. The preliminary model was fitted and checked for presence of autocorrelation and partial autocorrelation by using the Durbin Watson test. In addition, the augmented Dickey Fuller test for stationarity was also conducted on the outcome variable (Table 2). Results from the autocorrelation test for ANC (Figure 2a) showed a significant spike at lag 1 and decreases after a few lags, implying an autoregressive term in the data. The partial autoregressive function showed that the order of the autoregressive term was 1, and therefore, we modelled our series using autoregression (AR) (1). From the autocorrelation test for delivery care (Figure 2b), there were correlations at the first and second lags, followed by correlations that are not significant. This implies that the series had a moving average term. We observed a significant lag of 10 which implied that our series had order 2 AR process and moving average of order 10 [autoregressive moving average (2,10)].

**Table 2:** Test for stationarity

Outcome variable	Test statistic	1% critical value	5% critical value	10% critical value	*P*-value
Proportion of facility births	0.926	−3.75	−3.000	−2.630	0.9934
Proportion of women with four or more ANC visits	−2.147	−3.75	−3.000	−2.630	0.2260

#### Initial and final analysis

Initial segmented regression analysis was using the Newey–West model. The analysis was done based on residence (urban/rural) and wealth (upper/lower). Results from the autocorrelation test for delivery care in urban Zambia showed no autocorrelation at lags 1 and 3. Similarly, the autocorrelation test for delivery care in rural Zambia showed that there was no autocorrelation at lag 1. When we disaggregated the data by wealth, the results showed autocorrelation at lags 4 and 10 for the upper wealth quintile. For the lower wealth quintile, there was no serial correlation even at lag 1. For ANC, the results show autocorrelation at lag 3 for both urban and rural Zambia, and serial correlation for both the lower and upper wealth quintiles at lag 3. To correct all remaining serial correlation, we implemented the generalized least squares model, based on Prais–Winsten procedure.

### Analytical approach—survey data

Prais–Winsten segmented regression analysis was used to assess trends in ANC coverage and deliveries at health facilities before, during and after PBC. Segmented regression analysis was used because it is helpful in evaluating population-level effects in interrupted time series data, and it has the ability to control for secular trends and serial correlation (Wagner *et al.*, 2002; Linden, 2015). The data that were used for the analysis was ordered as a time series and a number of observations were available in both the pre-intervention and post-intervention periods to make a valid analysis (Wagner *et al.*, 2002; Campbell *et al.*, 2009; Wagenaar *et al.*, 2016). This was stratified by residence (rural and urban) and by wealth (upper two and lower two). For deliveries, observations were available in the pre-intervention, intervention and post-intervention periods; whereas for ANC the observations were only available during and after the intervention, because the wealth variable was not included in the 1992 round of DHS. Therefore, for deliveries, we compared the periods before, during and after PBC whereas for ANC we compared the periods during and after PBC.

The procedure that was used to prepare the data for analysis also conforms to suggested guidelines for analysis of time series data (Lagarde, 2012). After the data were ready for analysis, we used STATA version 15.1 to run the analysis. The STATA command for segmented regression analysis assumes a linear relationship between time and the outcome within each segment, and fits a least-squares regression line to each segment of the independent variable and time (Wagner *et al.*, 2002). For intervention status *j* and *k*, at time point *t*, the outcome is estimated with the following equation:

$$Y_{t}=\beta_{0}+\beta_{1}.T_{t}+\;\beta_{2}.X_{t}+\;\beta_{3}.{X_{t}.T}_{t}+\varepsilon_{t}$$

Where $Y_{t}$ is the aggregated outcome variable (ANC or delivery care), $T_{t}$ is time since the start of the study, $X_{t}$ is a dummy variable representing the intervention (pre-intervention = 0, otherwise = 1), $X_{t}T_{t}$ is an interaction term. The coefficients $\beta_{0}$ is the starting level of the outcome, $\beta_{1}$is the trend of the outcome before PBC, $\beta_{2}$is the change in level of the outcome in the period immediately following PBC, compared with pre-intervention levels and $\beta_{3}$ is the difference between pre- and post-intervention trends. For ANC, the equation was implemented with one interruption (after the end of PBC), whereas for delivery care we placed two interruptions (before and after PBC). We did the analysis by residence and wealth.

## Results

### Implementation process

PBC in the health sector in Zambia was part of the health reforms of 1991. The main features of the health reforms are articulated in a policy framework—the National Health Policies and Strategies (Ministry of Health, 1991). Prominent in this policy framework was the desire to build effective leadership, accountability and partnerships by decentralizing health service delivery (Ministry of Health, 1991), and establishment of the Sector-Wide Approach (SWAp) co-ordination mechanism in 1993 aimed at improving aid effectiveness (Chansa, 2009). As part of the health reforms, a purchaser-provider split was initiated by limiting the role of the Ministry of Health to policy development and advocacy, strategic planning, resource mobilization and allocation, and purchasing (Kalumba, 1997). For service delivery, an autonomous body—the CBoH—was created in 1996 to execute this function (Bossert *et al.*, 2003). Working collectively with the Zambian government, development partners were also instrumental in establishing the CBoH and provided a lot of financial and technical support. The responsibilities of the CBoH were contracting district and hospital boards to provide health services; regulating health services; performance assessment; and human resource management (Ministry of Health, 2004a).

Creation of the CBoH and use of PBC as a financing mechanism were backed by the 1995 National Health Services Act Cap 315 of the laws of Zambia (Bossert *et al.*, 2003). This paved the way for managerial and financial autonomy at district and hospital levels to be a central facet of the health reforms. As observed by Kalumba (1997):

*The Ministry [of Health] must now create conditions conducive to service delivery at all levels. With the new [national health services] Act, the Ministry of Health headquarters has become a purchaser of services provided by independent and semi-independent providers through the CBoH* (p. 18).

To operationalize the policy framework, the Ministry of Health developed the first National Strategic Health Plan in 1994 covering the period 1995−1997 (Lake and Musumali, 1999).^3^ In addition, a basic healthcare package of cost-effective services to be provided at primary health care level aimed at saving more lives with the available resources was also developed (Kalumba, 1997). Building on the national health strategic plan and basic healthcare package, detailed annual action plans and budgets were prepared by all the 72 districts which were in Zambia at that time as a requirement to receiving funding (Ministry of Health, 2000). The action plans were also the basis on which performance contracts between the CBoH and the district health boards were entered into (Ministry of Health, 2000).

*The fact that the action plan is the background for funding and for signing the performance contract, makes it a powerful incentive to develop a plan. The question is how it is adhered to in actually implementing activities during the year planned* (Ministry of Health, 2000: p. 48).

For PBC to work, new systems for planning, reporting, financing and expenditure control were put in place (Lake and Musumali, 1999; Bossert *et al.*, 2003). Key to the implementation process was direct financing to districts and bottom-up planning and management. Financial resources from the government and development partners were pooled together into what was termed as a ‘district basket’ and used to finance the provision of primary healthcare services at district level (Lake and Musumali, 1999). Annually, all the four main implementing entities at district level (communities, health posts, health centres and district hospitals) would prepare action plans and budgets and submit them to the district health management teams where they would be reviewed, aggregated and sent to District Health Boards for review and approval (Bossert *et al.*, 2003). Afterwards, the consolidated action plans and budgets would be sent to the CBoH for final endorsement (Bossert *et al.*, 2003). To ensure adherence to the approved annual action plans and health sector performance benchmarks, the health sector committee (comprising representatives from government, donors, non-governmental organizations (NGOs), civil society and other stakeholders in the health sector) used to meet quarterly^4^ to review progress on health service delivery and financial management (Lake and Musumali, 1999; Bossert *et al.*, 2003; Chansa, 2009).

About 4 years after the establishment of the CBoH, there were concerns that the CBoH was focusing too much on administrative and management procedures rather than on health service delivery (Ministry of Health, 2000); particularly for using process indicators to monitor performance instead of health output indicators (Ministry of Health, 2004a). Secondly, to incentivize performance, the health sector committee was mandated to approve the next tranche of funding only for districts with satisfactory financial and progress reports (Lake and Musumali, 1999). However, this principle was usually not followed, and funding was often approved for all the 72 districts regardless of performance. This problem was compounded by the absence of guidelines and/or clauses on sanctions for non-performance in the contracts (Ministry of Health, 2004b). This made it difficult for the CBoH to penalize the districts for poor performance.

*Currently, the contractual arrangements with the districts describe the activities to be included in the contract; these contracts, however, underplay issues of performance/sanctions for non-performance. By splitting the provider/buyer role, the Ministry of Health/CBoH has made an important first step towards a culture of performance. However, without defining incentives/sanctions to [non]performance, providers cannot yet be held accountable for their performance* (Ministry of Health, 2004b, p. 15).

Towards the end of 2003, calls for the abolition of the CBoH started emerging. It was argued that significant amounts of financial resources were being provided to the CBoH but the resources were being used for systems development rather than for improving health service delivery (Ministry of Health, 2004a). Further, it was also felt that the CBoH was spending a lot of financial resources at the centre rather than on the districts and health facilities (Chansa, 2009). However, the key issue was failure to transfer health workers from the civil service to the health management boards (Ministry of Health, 2004a). The process of delinking health workers from the civil service was halted by a high court injunction until demands by the Trade Unions were fulfilled, i.e. payment of full terminal benefits to all health workers who opted to leave the civil service to join the health management boards (Ministry of Health, 2004a). However, the government failed to raise the K400 billion (unrebased Zambian currency) or US$331 million (in 1996 current US$terms) that was required to pay terminal benefits to all the affected health workers (Ministry of Health, 2004a). Thus, the delinkage process was discontinued and this contributed to a situation whereby some health workers were employed by both the health management boards (or CBoH) and the civil service. This contributed to high expenditure on salaries and wages in the health sector, particularly at the centre (Chansa, 2009).

Lastly, although systems for implementing PBC were developed, there were some challenges in implementing the provider-purchaser split between the Ministry of Health and the CBoH. Functions for the Ministry of Health were not sufficiently stratified from those of the CBoH and this led to duplication of roles and responsibilities (Ministry of Health, 2004a). And although the CBoH put in place a number of systems to execute its functions, the Ministry of Health failed to develop systems to execute its core business as purchaser (Ministry of Health, 2004a). Eventually, the Ministry of Health became marginalized and the CBoH assumed the dual role of purchaser and provider (Ministry of Health, 2004a). By the end of 2004, development partners established firm relations with the CBoH and they could disburse funds under their own conditions for procurement, accounting and reporting without consulting the Ministry of Health (2004a). The relationship between the Ministry of Health and the CBoH was also undermined by fact that the two institutions were housed in the same building. Given the above factors, the CBoH was abolished by Parliament in April 2006 and its functions were taken over by the Ministry of Health.

### District-level expenditure

**Figure 3:**
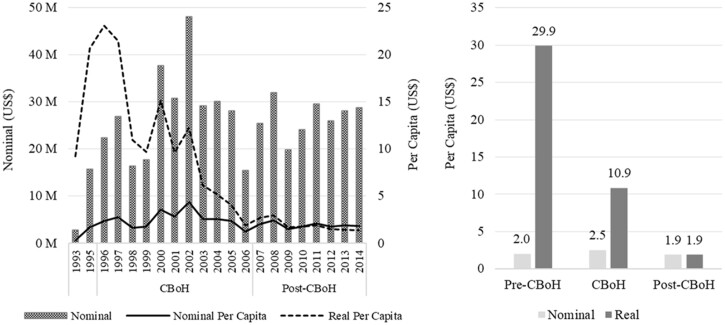
Trends in total and per capita health expenditure at district level (US$). *Source:* Authors’ construction from Ministry of Health data.

To effectively implement planned activities in the health sector and particularly at district level; the government pledged to be committing 13% of the total government budget to the health sector and to invest 20% of the government health sector budget at district level (Kalumba *et al.*, 1994). This commitment was partially met as government expenditure on health as a share of total government expenditure increased from 10.5% before the PBC era to 11.3% during the PBC era (Chansa, 2009). At district level, development partners complemented government’s efforts by providing significant amounts of financial resources; and this led to a nominal increase in the total government and donor expenditure at district level during the PBC era. In nominal terms, total expenditure on operational/service delivery activities^5^ through the district ‘basket’ funding modality increased from an annual average of US$9.2 million before the introduction of PBC to an annual average of US$27.5 million during the PBC era (Figure 3). After the PBC era, total expenditure on operational/service delivery at district level declined to US$26.7 million on average per year (Figure 3). However, in real terms (2010 terms), total expenditure on operational/service delivery activities at district level declined from US$134.4 million on average per year before the PBC era to US$112.2 million on average per year during the PBC era, and US$26.2 million on average per year after the PBC era.

In per capita terms, there was a nominal increase in total expenditure on operational/service delivery activities at district level from US$2.00 per person per year before the PBC era to US$2.50 during the PBC era and a decline to US$1.90 after the PBC era (Figure 3). A detailed review of the individual years during the PBC era shows an increasing trend in nominal per capita expenditure between 1996 and 2002, and a declining trend from 2003 to 2006 with a low of US$1.25 in 2006. In real terms (2010 terms), per capita total expenditure on operational/service delivery activities at district level declined from US$29.90 per person per year before the PBC era to US$10.90 and US$1.90 during and after the PBC era, respectively (Figure 3). For the individual years, there is an increasing trend in real per capita expenditure before the PBC era from US$9.20 per person per year in 1993 to US$23.10 in 1996, and a declining trend throughout the PBC era with a low of US$1.90 in 2006 (Figure 3). The declining level in the annual per capita expenditure at district level during the period under review could be attributed to an increase in population growth, fluctuations in the US$−Zambian Kwacha (ZMW) exchange rate, and high levels of inflation. Zambia’s population grew at a rate of 2.8% per annum during the period 2000−10 (Central Statistical Office, 2012), whereas inflation in Zambia was estimated at 18% on average per annum over the period 1995−2014 (Roger *et al.*, 2017). Further, the level of district health spending during the period under review was hampered by fluctuations in the exchange rate (Chansa *et al.*, 2018).

### Time series analysis

**Figure 4:**
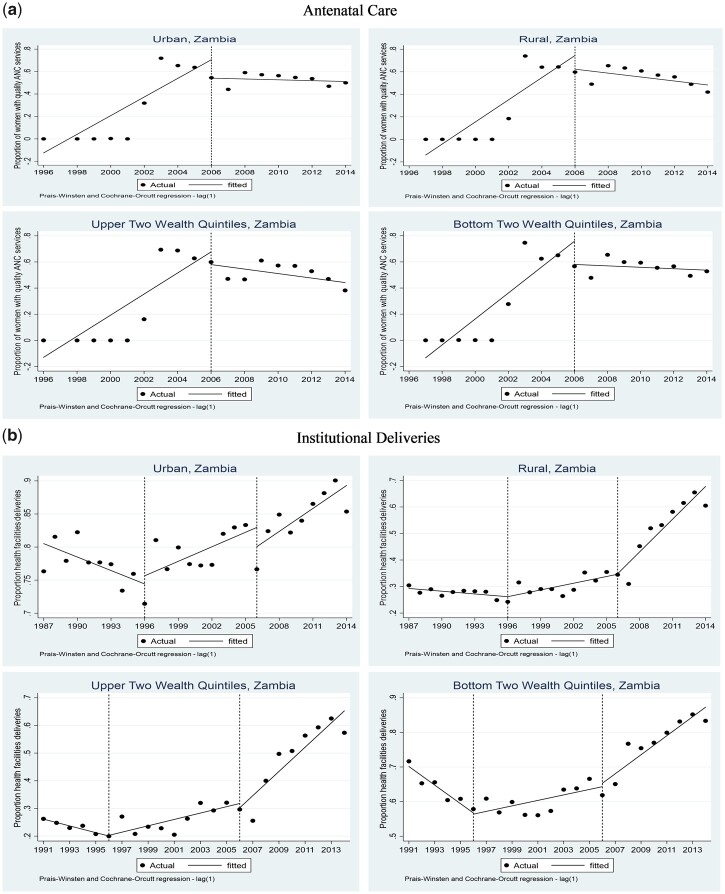
ANC and deliveries at health facilities by residence and wealth status. *Source:* Authors’ construction from DHS data.

Level and trend analyses of coverage of the two (2) main variables of interest are presented in Figure 4 and Table 3. For ANC services, the level before the PBC era could not be established due to lack of data. The trend analysis by residence (urban/rural) during the PBC era (1996−2006) shows that there was an 8% mean rate increase in the proportion of women who had four or more ANC visits in urban areas over the years (*P* < 0.01); but there was no significant change in rural areas (Table 3). After the end of PBC, there was a 17% decrease in the proportion of women who had four or more ANC visits in rural areas (*P* < 0.01); but the results for urban areas were not statistically significant (Table 3). However, there was a decrease in the trend for the proportion of women who had four or more ANC visits after the PBC era (9% on average) in urban areas (*P* < 0.01); whereas the results for rural areas were not statistically significant. With regards to wealth status, trend analysis during the PBC era showed an 8% mean rate increase in the proportion of women who had four or more ANC visits for both the lower and upper wealth quintiles over the years (*P* < 0.01). After the PBC, there was a decrease in the trend in ANC coverage in the upper two wealth quantiles at 10% (*P* < 0.01); and in the lower two wealth quantiles at 9% (*P* < 0.01; Table 3).

**Table 3:** Model results for the Prais–Winsten Regression Analysis

Indicator	Residence		Wealth	
	Urban	Rural	Upper	Lower
ANC—four or more visits				
1996 (start of intervention)				
Trend during intervention	0.08***	0.09	0.08***	0.08***
2006 (end of intervention)				
Level change after end of intervention	−0.20	−0.17***	−0.21*	−0.19
Trend change after end of intervention	−0.09***	−0.11	−0.10***	−0.09***
Post-intervention trend	−0.004	−0.02	−0.013	−0.006
Model summary				
*F*-statistic	16.24***	15.98***	12.83***	18.09***
*R*-squared	0.57	0.64	0.57	0.64
Health facility deliveries				
Pre-intervention trend	−0.01**	−0.003*	−0.14***	−0.025***
1996 (start of intervention)				
Level change following intervention	0.02	0.00	0.04	0.02
Trend change following intervention	0.01***	0.11***	0.02***	0.32***
Post-intervention trend	0.01*	0.009***	0.01**	0.01**
2006 (end of intervention)				
Level change after end of intervention	−0.00	0.04	0.02	0.06
Trend change after end of intervention	0.00	0.03***	0.03**	0.02**
Post-intervention trend	0.01***	0.04***	0.04***	0.02**
Model summary				
*F*-statistic	28.30***	48.57***	43.51***	86.07***
*R*-squared	0.93	0.94	0.90	0.93

* *P* < 0.1, **P < 0.05, ***P < 0.01.

For health facility deliveries, the results indicate that before PBC was introduced, the proportion of deliveries at health facilities was declining at a rate of 1% (*P* < 0.05) and 0.3% (*P* < 0.1) on average in urban and rural areas, respectively (Table 3). When PBC was introduced, there was no significant level change in the proportion of deliveries at health facilities in both urban and rural areas (Table 3). However, there was an 11% (*P* < 0.01) mean rate increase in health facility deliveries in rural areas as compared with a 1% (*P* < 0.01) increase that was observed in urban areas (Table 3). After the PBC era, the positive trend continued in rural areas (*P* < 0.01) but the results for urban areas were not statistically significant. For wealth status, before the PBC, the proportion of deliveries at health facilities was declining at a mean rate of 14% (*P* < 0.01) among women in the upper two wealth quintiles as compared with 3% (*P* < 0.01) among women in the lower two wealth quintiles (*P* < 0.01). When the PBC was introduced, there was no level change across the wealth quintiles but there were huge differences in the trends. There was a 32% (*P* < 0.01) mean rate increase in health facility deliveries among women in the lower two wealth quintiles as compared with the 2% (*P* < 0.01) that was observed among women in the upper two wealth quintiles (Table 3). After the PBC, the mean rate increase in health facility deliveries across the wealth quintiles was basically the same.

## Discussion

This study examined the association between PBC and equity of access to maternal health services in Zambia over the period 1996−2006. Results from the study show that contracting of district health services was an integral part of the 1992 health reforms in Zambia. The starting point was decentralization of health service delivery by devolving the key functions of the Ministry of Health to districts and autonomous hospital management boards, and this provided a conducive environment for separation of power and contracting with the district health boards. Most importantly, PBC and the overall process of managing health services were anchored by a legal framework—the National Health Services Act of 1995. This is similar to New Zealand where the contracting environment was shaped by a legal framework (Ashton *et al.*, 2004). This underscores the importance of formalizing health system-wide PBC arrangements through an Act of Parliament.

Other than the decentralization of health services and the legal framework, high-level political support, articulation of the health vision through the 1992 policy framework, and involvement of development partners and other stakeholders through the SWAp also helped to shape the implementation of PBC in Zambia. This is important because the context in which PBC is implemented and the design features of the interventions are key factors for success (Liu *et al.*, 2007). As observed in Zambia, delivery of an overarching policy framework, multi-year and annual planning, and development of systems for financial and performance monitoring contributed to increased transparency and accountability in the management of resources. However, our results show that there were a number of problems that were associated with the provider-purchaser split. Foremost, the relationship between the Ministry of Health and the CBoH was hampered by failure to fully separate the functions of the two institutions and this led to duplication of roles and responsibilities; and eventually the CBoH assumed the dual role of purchaser and provider. This situation was undesirable because a good relationship between contracting parties is key to successful contracting (Ashton *et al.*, 2004). Secondly, the CBoH was criticized for concentrating too much on systems development rather than service delivery, failure to incentivize performance and not having in place guidelines to sanction poor performance. However, with the district as the lowest unit at which the contracts were enforced, we assume that it would have been difficult to punish all the health facilities in the non-performing districts by suspending or withholding funding. This probably explains why the CBoH sought to conduct follow-up audits or monitoring visits to non-performing districts rather than penalizing them (Lake and Musumali, 1999). On the other hand, the failed delinkage process contributed to high expenditure on salaries and wages, and marginalization of the role of health management boards and the CBoH in controlling workers in the health sector.

Our study also looked at the level of expenditure on operational/service delivery activities at district level. This is important because it was anticipated that the resource envelope for the health sector would expand whereas equity in financing would be enhanced during the PBC era. As observed by Kalumba *et al.* (1994), the health reforms were expected to facilitate an increase in the resource envelope for the health sector from government and development partners. However, the results show that in real terms, per capita health expenditures at district level declined consistently during the PBC era. This was mostly due to a rising population, fluctuations in the US$−ZMW exchange rate and high inflation. This implies that to achieve better health outcomes health authorities had to be prudent and efficient in resource use. Considering that the percentage of pregnant women accessing ANC services and delivering at health facilities increased despite lower per capita expenditure during the PBC era suggests that PBC is a cost-efficient intervention.

To substantiate the above assertion, the results show an association between PBC and increase in the proportion of women who had four or more ANC visits for both the lower and upper wealth quintiles during the PBC era (1996−2006). Further, PBC was associated with an increase in the proportion of deliveries at health facilities in both urban and rural areas, and for both lower and upper wealth quintiles. However, the correlation was significantly higher in rural areas and for the lower wealth quintiles. These results are consistent with findings by Bhushan *et al.* (2002) who observed that districts that had been contracted-in and contracted-out in Cambodia provided higher benefits to the poor as compared with the controls. Mahmud *et al.* (2002) cited by Liu *et al.* (2007) also reports that contracting NGOs to provide primary healthcare services for urban slums in Bangladesh had contributed to a significant improvement in access by the urban poor. However, the emphasis in these two studies was on contracting-out primary healthcare services to NGOs whereas in Zambia an autonomous public institution (CBoH) was contracted-in. In nominal terms, the intervention in Zambia seems to have been more cost-efficient than in Cambodia. For Cambodia, total per capita spending was estimated at US$2.60 per capita in contracted-in districts and US$2.90 per capita in contracted-out districts (Bloom *et al.*, 2006) which are relatively higher than the US$2.50 per capita spending in Zambia during the PBC era. The fact that the intervention in Zambia was system-wide and countrywide suggests that it was considerably less expensive.

The other added value of our study is that it looks at the correlation of PBC to the variables of interest over a long period of time, i.e. 10 years for the intervention, and a number of years before and after the intervention. Most of the studies on PBC have only evaluated the immediate or short-term effects associated with PBC which is contrary to conventional norms for analysing the impact of a policy change (Bernal *et al.*, 2017; Lagarde, 2012). Our study shows that PBC was associated with improved coverage of maternal health services with positive trends continuing after the intervention. Furthermore, results from the 2013−14 DHS (Central Statistical Office *et al.*, 2014) show that the maternal mortality ratio (MMR) increased from 649 deaths per 100 000 live births over the period 1990−96 (before the PBC era) to 729 deaths per 100 000 live births over the period 1996−2001 (early period of the PBC era). The MMR then fell to 591 deaths per 100 000 live births over the period 2001−07 (later period of the PBC era), and 398 deaths per 100 000 live births over the period 2007−13 (after the PBC era). This suggests that improvements in the coverage of maternal health services were associated with better maternal health outcomes.

Notwithstanding the above, there were other pieces of health reforms that were implemented after the intervention (i.e. removal of user fees in April 2006, 2007 and 2012) which could have also contributed to the sustained positive correlation. Nonetheless, it is important to note that the post-intervention trend for health facility deliveries remained the same but intensified after the PBC whereas there was an inverse trend in ANC coverage. This implies that PBC had a bearing on ANC coverage and health facility deliveries. Secondly, even after abandoning PBC and restructuring the health sector a number of times; the ‘performance culture’ that was instilled during the PBC era has been sustained (Chilufya and Kamanga, 2018). For instance, the Ministry of Health has adopted the implementation structures which were left behind by the CBoH and has been implementing contracted-in results-based financing (RBF)^6^ projects in several parts of the country since 2008 (Friedman *et al.*, 2016). Thus, though PBC is relatively unpopular nowadays, its elements are still being implemented in Zambia where it has laid the foundation for RBF. As observed in some studies, PBC was mostly used in developing countries in the 1990s (Mills and Broomberg, 1998) and the evolution to RBF since 2002 (Fritsche *et al.*, 2014) demonstrates that PBC is still a central part of the management of health systems in developing countries.

### Limitations of the study

Firstly, it is important to highlight that the observed relationships are expressions of correlation and not causation. Secondly, the main model used for this analysis (segmented regression analysis) assumes linearity in outcomes within each segment but in reality, the outcomes could follow non-linear trends (Wagner *et al.*, 2002). Thirdly, the short-time period did not provide adequate data points, which may have affected the predictive power of the model. However, since data on utilization only complemented the financial and qualitative data—which also show that PBC is a favourable intervention—we are reasonably satisfied that to a large extent our conclusions are valid. Fourth, a number of policy interventions such as decentralization or devolution of health services and introduction of the health SWAp took place during the CBoH era. However, these policy measures were prerequisites for a functional PBC and provided the working environment for the implementation of PBC in Zambia. Lastly, after the abolition of the CBoH in 2006, user fees were abolished in 2006, 2007 and 2012. This could also have led to increased utilization of health services after the CBoH era. However, maternal health services were being provided free of charge during the CBoH era and when user fees were in place. Thus, any potential bias is expected to be minimal.

## Conclusion

Based on the evidence presented in this article, we conclude that contracting-in a quasi-autonomous public institution to manage health services at district level has the potential to improve equity of access to maternal health services. The experience in Zambia suggests that high-level political support, a legal framework, and having in place a policy framework that guides multi-year and annual planning processes are key to the success. In addition, decentralizing health service provision and collective planning and implementation with all key stakeholders are also important. The study further shows that PBC is a cost-efficient and sustainable policy reform. However, as with any major reform, there are some implementation challenges associated with PBC. The study calls for policymakers to clearly stratify roles and responsibilities between contracted parties, and to comprehensively evaluate the impact of health system reforms before terminating them.

## Footnotes

1 This excludes expenditure on salaries and wages, drugs and medical supplies, and major capital investment projects which were being funded directly from the Ministry of Health headquarters.

2 Obtained from www.zamstats.gov.zm.

3 During the PBC era, three strategic plans were developed the others covering the periods 1998 − 2000 and 2001−05.

4 In 2004, the number of review meetings were reduced from quarterly to bi-annual. The Health Sector Steering Committee was renamed the Health Sector Advisory Group in 2006.

5 This excludes drugs and human resources (salaries and wages).

6 In RBF, health facilities are usually contracted and the performance outputs that are used are often at health facility level. Further, fee-for-service is the most commonly used provider payment mechanism in RBF programmes. In addition, RBF performance incentives are directed at health facilities, individual providers and in some cases beneficiaries. In PBC, private or public organizations are contracted whereas budgeted funds are transferred to the contracted entity as a lump sum when performance targets are met. In most cases, performance incentives in a PBC programme are only at institutional level.
